# Adverse events from topical corticosteroid use in chronic hand eczema — Findings from the Danish Skin Cohort

**DOI:** 10.1016/j.jdin.2023.11.004

**Published:** 2023-12-03

**Authors:** Alexander Egeberg, Christoph Schlapbach, Jeanette Halskou Haugaard, Lea Nymand, David Thein, Simon Francis Thomsen, Jacob P. Thyssen

**Affiliations:** a Department of Dermatology and Venereology, Bispebjerg Hospital, Copenhagen, Denmark; b Department of Clinical Medicine, Faculty of Health and Medical Sciences, University of Copenhagen, Copenhagen, Denmark; c Department of Dermatology, Inselspital, Bern University Hospital, University of Bern, Bern, Switzerland; d Department of Biomedical Sciences, Faculty of Health and Medical Sciences, University of Copenhagen, Copenhagen, Denmark

**Keywords:** adverse events, chronic hand eczema, Danish Skin Cohort, side effects, topical corticosteroids

## Abstract

**Background:**

Topical corticosteroids (TCS) are used to treat most patients with chronic hand eczema (CHE), but knowledge about TCS-related adverse events in CHE is limited.

**Objectives:**

To investigate patient-reported adverse events to TCS in CHE patients.

**Methods:**

Data on adverse events related to TCS use in patients with CHE were analyzed from the Danish Skin Cohort; a prospective survey of a hospital cohort. We assessed patients’ knowledge about TCS use and adverse event risks, and preference of TCS versus a nonsteroidal topical alternative.

**Results:**

Of 724 adults with CHE (64.0% women; mean age 57.5 [standard deviation 12.8] years), 64.1% reported skin atrophy, 41.4% cracks/fissures, 23.9% bleeding, 45.9% pain/stinging sensation, 40.0% reduced hand dexterity, and 40.2% worsening of CHE signs or symptoms from using TCS. We observed CHE-severity-dependent associations (all groups; *P* < .0001). Most patients (76.4%) would prefer a nonsteroidal option, 10.9% were neutral/indifferent, and 12.7% would prefer TCS for CHE. The median numerical rating scale-score (ranging from 0 to 10) was 10 (interquartile range 6-10) for preferring a nonsteroidal topical treatment.

**Limitations:**

Differences across TCS formulations were unexplored.

**Conclusion:**

TCS-related cutaneous adverse events were common. There is a desire from patients for novel steroid-free topical alternatives for CHE treatment.

**Capsule Summary**

• Topical corticosteroids are frequently used for acute and long-term treatment of chronic hand eczema, but robust data on adverse events in these patients remain lacking.
• Adverse events such as skin atrophy, cracks/fissures, pain/stinging, reduced hand function, and worsening of chronic hand eczema signs or symptoms were all frequently occurring adverse events.
• The majority of patients would prefer a nonsteroidal topical therapy for treatment of their hand eczema.

## Introduction

Hand eczema is a common condition that affect up to 10% of adults from the general population over a given 12-month period.^1^^,^^2^ Although most patients have mild and short-lasting symptoms, some develop chronic hand eczema (CHE), defined as an eczematous process on the hands and or wrists that persists for >3 months, or that demonstrates >2 relapses within a single year.^3^ Hand eczema is mostly elicited by repeated or continuous exposure to skin irritants and/or contact allergens, which may be difficult to avoid both at work and at home.^4^ In a Swedish 15-year follow-up study of 868 people with hand eczema at baseline, originally sampled from the general population, 66% reported periods of hand eczema and 44% had symptoms within the past 12 months, emphasizing the chronicity of the condition.^5^ Predictive factors for hand eczema 15 years later included a history of atopic dermatitis, early onset of hand eczema, and extensive eczema at onset.^6^

Treatment of hand eczema includes avoidance of trigger factors, and skin protection including education about daily use of emollients.^3^ Acute or prolonged treatment with antiinflammatory therapies such as topical corticosteroids (TCS) is often necessary and this is typically done with 2 to 3 weekly applications in the maintenance phase, since more frequent TCS dosing in the long run causes skin atrophy, fissures, and pain.^7^, ^8^, ^9^ TCS may not be effective in treatment of irritant contact dermatitis,^10^^,^^11^ which affects most patients with CHE, in turn leading to a poor prognosis for some. There is currently little insight into side effects of TCS use in patients with CHE as well as patient preferences regarding topical treatments.^12^

Due to the protracted nature of CHE treatment, further information and quantification of potential adverse effects of TCS among patients with CHE is warranted. We therefore examined patient knowledge about, and prevalence of, TCS-related adverse events as well as patients’ treatment preferences in a population-based cohort of adult patients with CHE in Denmark.

## Materials and methods

The study was registered at the Capital Region’s inventory (Videncenter for Dataanmeldelser, ref. P-2021-386). All participants gave written informed consent to participate and allowed processing of personal information. This constitutes the necessary legal requirements, and ethical approval is not required for this type of study in Denmark.

The Danish Skin Cohort is a prospective cohort comprising adults with a number of skin diseases, including CHE. Patients are identified, and their diagnoses are verified, by dermatologists from academic hospital centers as well as from a number of private dermatology clinics in Denmark. Design and data collection methods for the Danish Skin Cohort have previously been described in detail.^13^ Briefly, patients with dermatologist-verified CHE in the Danish Skin Cohort were interviewed in a structured manner through a secure digital system, with clinical photographs being made available as appropriate. Patients’ severity of CHE was defined based on the photographic guide by Coenraads et al,^14^ but since the present study examined “*worst ever* severity of CHE,” 4 groups were used, with “Clear or almost clear” representing the mildest group rather than “Completely clear.”

Between June 23, 2023, and July 14, 2023, patients with CHE from the Danish Skin Cohort reported data regarding adverse events specifically relating to their use of TCS for their CHE. Thus, events (eg, cracks/fissures) occurring due to CHE itself were not included as an adverse event. Questions included their knowledge about use and adverse events risks before using TCS, and whether they had experienced skin atrophy, cracks or fissures, bleeding, skin pain or stinging, reduced function of their hands, worsening of CHE or skin barrier, or skin bruising, respectively, as a result of using TCS. Patients were furthermore asked to rate the extent/severity of these adverse events, and were also asked to rate on a numerical rating scale (NRS; 0 = completely disagree; 10 = completely agree) how much they agreed or disagreed with the following statement: “*If there was a nonsteroidal topical treatment for my chronic hand eczema, I would prefer this over TCS*.”

### Statistical analysis

Summary statistics were created and presented as frequencies with percentages for categorical variables and means with standard deviations (SDs) for continuous variables. Furthermore, interquartile ranges (IQRs) were estimated for nonnormally distributed continuous outcome variables. The Cochran-Armitage test was used to test for trend across CHE severity strata. *P* values < .05 were considered statistically significant. Analyses were performed using and Stata software version 18 (StataCorp).

## Results

Out of 1,296 patients with CHE in the from the Danish Skin Cohort, 724 provided information about adverse events of TCS when used for their CHE. Mean age was 57.5 (SD 12.8) years and mean age of CHE onset was 36.9 (SD 17.1) years. There was a female predominance (64.0%, *n* = 463), and the majority of patients were either current (17.4%, *n* = 126) or former smokers (47.7%, *n* = 345) (Table I). A total of 586 patients (80.9%) had data on CHE severity; 20.3% (*n* = 199) reported clear or almost clear CHE, 27.8% (*n* = 163) had moderate CHE, 26.5% (*n* = 155) had severe CHE, and 25.4% (*n* = 149) had very severe CHE. Among patients with CHE, 30.0% (*n* = 200) had a history of atopic dermatitis. Among 368 (50.8%) patients who had active disease within the last 12 months, 70.1% (*n* = 258) reported symptoms persisting for >3 months, and for 88.6% (*n* = 326) symptoms returned twice or more within these 12 months. In total, 349 (94.8%) of these patients had either persistent symptoms for >3 months, or symptoms returning ≥2 times within the last 12 months. Mean time from first-ever episode of hand eczema was 21.1 (SD 13.5) years.

**Table I:** Characteristics of the study population

Characteristic	Chronic hand eczema (*n* = 724)
Age, y, mean (SD)	57.5 (12.8)
Sex, *n* (%)	
Female	463 (64.0)
Male	261 (36.0)
Age at CHE onset, mean (SD)	36.9 (17.1)
Smoking, *n* (%)	
Current smoker	126 (17.4)
Former smoker	345 (47.7)
CHE severity, *n* (%)∗	
Clear or almost clear	119 (20.3)
Moderate	163 (27.8)
Severe	155 (26.5)
Very severe	149 (25.4)
Received information about how to use TCS, *n* (%)	
From prescribing physician	545 (75.3)
From nurse	73 (10.1)
From pharmacy	114 (15.8)
Received information about risk of TCS side effects, *n* (%)	
From prescribing physician	331 (45.7)
From nurse	56 (7.7)
From pharmacy	72 (9.9)
Online search	114 (15.8)

*CHE*, Chronic hand eczema; *SD*, standard deviation; *TCS*, topical corticosteroids.

∗ Data available for subset (*n* = 586) of patients.

### *A priori* knowledge about TCS use and adverse event risks

When asked whether patients received instructions on how to use their TCS (quantity and frequency of use), 75.3% (*n* = 545) patients reported that they were told by the physician that had prescribed the TCS, 10.1% (*n* = 73) were told by a nurse practitioner, and 15.8% (*n* = 114) were told by the pharmacist when filling the prescription. In total, 45.7% (*n* = 331) patients received information about potential risks of TCS adverse events from the prescribing physician, 7.7% (*n* = 56) were informed by a nurse, 9.9% (*n* = 72) were told by the pharmacist, and 15.8% (*n* = 114) reported that they searched online for safety information before using their TCS. When asked specifically about awareness of the risk of skin atrophy when using TCS for CHE, 345 (47.7%), 70 (9.7%), and 51 (7.0%) were told by the prescribing physician, nurse, and pharmacist, respectively, and 99 (13.7%) of patients had found this information through online search.

### Adverse events from use of TCS for CHE

**Fig 1:**
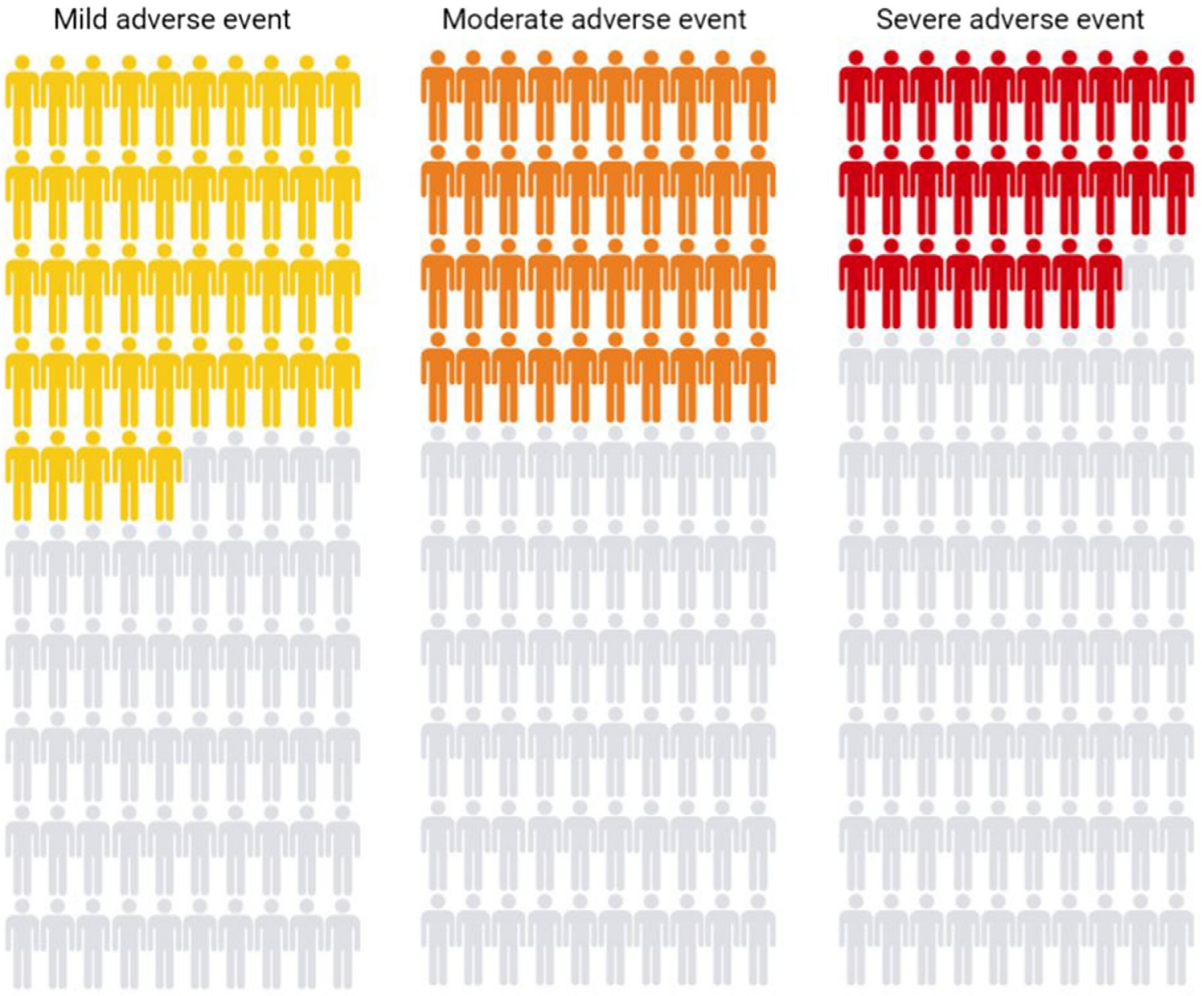
Proportion of patients reporting having had at least one mild (45%), moderate (40%), and severe (28%) adverse event from using topical corticosteroids for chronic hand eczema.

**Fig 2:**
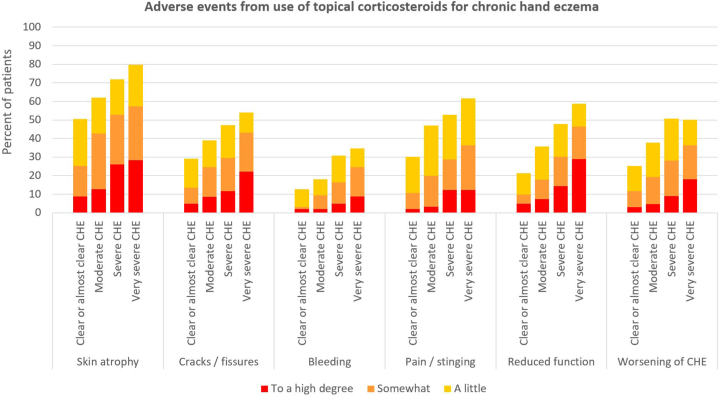
Adverse events from use of topical corticosteroids for chronic hand eczema.

A total of 614 patients (84.8%) reported having experienced at least one adverse event from use of TCS for their CHE. Stratified by patient-reported adverse event severity, 45.0%, 40.1%, and 28.3% of patients reported experiencing at least one mild, moderate, or severe adverse events, respectively (Fig 1). Bruising was relatively infrequent with use of TCS for CHE (5.4%, *n* = 39), however prevalence of bruising was higher for severe (5.2%) and very severe CHE (7.4%), than with clear or almost clear (4.2%) or moderate CHE (4.9%). Overall, 64.1% of patients had experienced skin atrophy, 41.4% had developed cracks or fissures, 23.9% had experienced bleeding, 45.9% had pain or stinging, 40.0% had reduced function of their hands, and 40.2% had worsening of signs or symptoms of their CHE from use of TCS. Stratified by severity of CHE, we observed consistent severity-dependent trends (all *P* < .0001) for the reported adverse events across all groups (Fig 2 and Supplementary Table I, available via Mendeley at https://data.mendeley.com/datasets/r7sg8t4y8w/1).

Among patients using TCS as maintenance therapy (ie, >2 times per week), 81.1% reported having experienced skin atrophy, 56.6% had developed cracks/fissures, 33.6% experienced bleeding, 57.8% had pain or stinging from using TCS, 53.5% had reduced function of their hands resulting from TCS use, and 54.7% felt that signs or symptoms of their CHE worsened as a result of applying TCS to their hands (Supplementary Fig 1, available via Mendeley at https://data.mendeley.com/datasets/r7sg8t4y8w/1).

### Patients’ preferences for a nonsteroidal topical treatment instead of TCS

**Fig 3:**
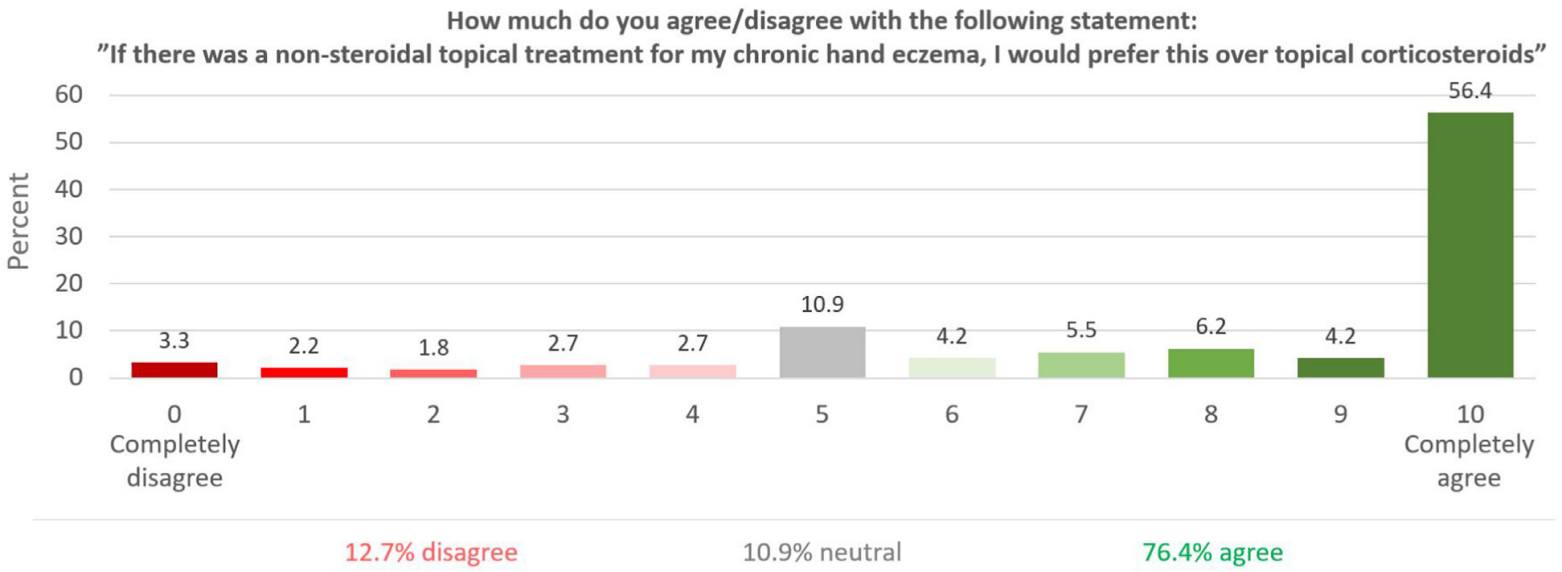
Patient-reported preference of a nonsteroidal topical treatment versus topical corticosteroids for treatment of chronic hand eczema.

When patients were asked to rate whether they would prefer a nonsteroidal topical treatment rather than TCS, the majority (76.4%) preferred a nonsteroidal option, 10.9% were neutral/indifferent, and 12.7% preferred a TCS (Fig 3). The median NRS-score was 10 (IQR 6-10), and more than half of patients (56.4%) reported that they “completely agreed” (NRS = 10), as opposed to only 3.3% who “completely disagreed” (NRS = 0). When limited to patients that had never experienced any adverse events of TCS (*n* = 110), 65.5% (*n* = 72) of patients stated that they would prefer a nonsteroidal topical treatment (median NRS = 8, IQR 5-10), including 52 patients (47.3%) who “completely agreed.” Conversely, 15.5% (*n* = 37) were neutral, and 19.1% (*n* = 21) disagreed.

## Discussion

In this study of 724 patients with CHE, >8 out of every 10 patients had experienced some degree of adverse events when using TCS, and 28.3% reported at least one adverse event as severe. Most patients had received some degree of information about dosage and adverse events risks from health care personnel before using TCS, and >3 out of every 4 patients said they would prefer a nonsteroidal topical treatment alternative to TCS.

TCS were introduced in the 1950s and incremental innovation has led to modern TCS being effective medications with a favorable safety profile as long as treatment complies with the correct dosing and frequency of application. Examples of adverse events with excessive quantities of TCS use in dermatological diseases includes risk of type 2 diabetes and osteoporosis.^15^, ^16^, ^17^ The overarching aim for physicians is therefore to prescribe sufficient amounts of TCS to reduce skin inflammation, but little enough that the skin barrier can still improve and heal following the negative effects of eczema and irritant exposure, and systemic exposure kept to a minimum.

Treatment of moderate-to-severe CHE with TCS represents a particular challenge for physicians and patients. Thus, since irritant or allergic contact dermatitis is involved in the etiopathogenesis of most cases, and it is difficult to avoid exposure to exogenous stressors including skin irritants and allergens on the hands during a normal day, both at work and at home,^4^ thus patients have a high risk of progressing to chronic disease. Those individuals that can rapidly avoid culprit skin exposures will have a more favorable prognosis, whereas those that are unable to avoid these are at higher risk of developing CHE.^18^ High, or even excessive, use of potent or very potent TCS may be installed in an attempt to reduce eczema severity, but this may ultimately lead to skin atrophy, fissures and pain as reported by the patients in this study. For this reason, dermatologists often recommend less frequent dosing of TCS, eg, twice weekly to reduce the risk. However, this will inevitably reduce the antiinflammatory need, and hand eczema may end up being a very chronic disease as previously reported.^5^

The desire for new steroid-sparing topical treatment alternatives is highlighted in our study, where >3 of every 4 patients reported preferring a nonsteroid topical treatment. Notably, even among TCS users that had never experienced adverse events from their treatment, more than half of patients reported that they would have preferred a nonsteroid option. Indeed, prescriptions of TCS are often accompanied by poor adherence due to steroid phobia, even in the absence of local adverse events, as well as insufficient treatment response in some patients,^19^ and the chronic and relapsing nature of CHE means that patients may need sustained, sometimes even lifelong, treatment of the disease. Such need for prolonged treatment poses a considerable worry about of adverse events and skin barrier impairment with use of TCS.^17^ Consequently, there remains a significant need for novel topical therapies for CHE, that can rapidly improve signs and symptoms, including pruritus, while limiting the risk of local or systemic adverse events. Topicals that can be used daily to reduce inflammation, and without the inherent risk of damaging the skin barrier such as potent TCS, would at least in theory represent a treatment solution with a greater chance of disease modification.

### Limitations

Several limitations apply to the present study. Although we examined a selection of adverse events known to be related to TCS, it is possible that patients may have experienced other types of adverse events (or have been unaware if the occurrence was related to their TCS treatment), and the prevalence of “any adverse event” may therefore potentially be somewhat underestimated. Importantly, data were self-reported by patients, and severity of adverse events is therefore a subjective measure, as opposed to an objective physician assessment of severity. Furthermore, although patients generally had received information about which frequency and quantity of TCS they should apply, the exact amount of applied TCS, and duration of treatment, remains unknown. Moreover, when patients are informed about a potential side effect risk by their health care practitioner, they may be more attentive to this and thus notice signs or symptoms that would otherwise have gone undetected, thus resulting in an overrepresentation of (particularly milder) adverse events. Lastly, although TCS in this study serves as an umbrella term for (typically potent or ultrapotent) TCS products used for CHE, there may be between-drug differences in the frequency of adverse events with certain compounds or formulations.

In conclusion, we found that the prevalence of TCS-related adverse events was high, especially among patients with moderate-to-severe CHE. Although patients received information about such risks from their health care practitioner, further risk mitigation strategies are warranted for optimal long-term management of patients with CHE. More than 3 out of every 4 patients expressed preference for a nonsteroidal topical therapy over TCS, emphasizing the need for novel topical alternatives for treatment of CHE.

## Conflicts of interest

Dr Egeberg has received research funding from Almirall, Pfizer, Eli Lilly, Novartis, Bristol-Myers Squibb, AbbVie, Janssen Pharmaceuticals, Boehringer Ingelheim, the Danish National Psoriasis Foundation, the Simon Spies Foundation, and the Kgl Hofbundtmager Aage Bang Foundation, and honoraria as consultant and/or speaker from Amgen, AbbVie, Almirall, LEO Pharma, Zuellig Pharma Ltd, Galápagos NV, Sun Pharmaceuticals, Samsung Bioepis Co, Ltd, Pfizer, Eli Lilly and Company, Novartis, Union Therapeutics, Galderma, Dermavant, UCB, Mylan, Bristol-Myers Squibb, McNeil Consumer Healthcare, Horizon Therapeutics, Boehringer Ingelheim, and Janssen Pharmaceuticals. Dr Schlapbach has received research funding from PPM Services/Nogra Group, and honoraria as consultant and/or speaker from AbbVie, Almirall, Incyte, Kiowa Kirin, Sanofi, LEO Pharma, Pfizer, Eli Lilly and Company, Novartis, and Bristol-Myers Squibb. Dr Thomsen has been a speaker or adviser for Sanofi, AbbVie, LEO Pharma, Pfizer, Eli Lilly, Novartis, UCB Pharma, Symphogen, UNION Therapeutics, Almirall, and Janssen Pharmaceuticals, and received research support from Sanofi, AbbVie, LEO Pharma, Novartis, UCB Pharma, and Janssen Pharmaceuticals. Dr Thyssen is an adviser for AbbVie, Almirall, Arena Pharmaceuticals, Coloplast, OM Pharma, Aslan Pharmaceuticals, Union Therapeutics, Eli Lilly & Co, LEO Pharma, Pfizer, Regeneron, and Sanofi-Genzyme, a speaker for AbbVie, Almirall, Eli Lilly & Co, LEO Pharma, Pfizer, Regeneron, and Sanofi-Genzyme, has received research grants from Pfizer, Regeneron, and Sanofi-Genzyme, and is currently an employee at LEO Pharma. Drs Haugaard and Thein and Author Nymand have no conflicts of interest to declare.
